# Acylated Ghrelin Mediates the Link Between *Akkermansia* Abundance and Insulin Resistance in Type 2 Diabetes

**DOI:** 10.1155/jdr/4051518

**Published:** 2025-08-05

**Authors:** Zhen Zhang, Xi Jiang, Li Zhang, Ying Feng, Jiahao Tang, Yuan Xu, Yunchong Guo, Peng Yun, Fangping Li

**Affiliations:** ^1^Department of Endocrinology, The Seventh Affiliated Hospital, Sun Yat-sen University, Shenzhen, China; ^2^Department of Clinical Laboratory, The Seventh Affiliated Hospital, Sun Yat-sen University, Shenzhen, China

**Keywords:** acylated ghrelin, *Akkermansia*, insulin resistance

## Abstract

**Aims:** This mediation analysis was aimed at examining whether ghrelin mediates the association between gut microbiota and glucose metabolic indices.

**Materials and Methods:** Fifty-five patients with newly diagnosed Type 2 diabetes mellitus (T2DM) and 40 healthy controls (HCs) were included. Serum ghrelin levels were measured by ELISA. Fecal 16S rRNA gene sequencing was performed, and gut microbiota differences were assessed using linear discriminant analysis effect size (LEfSe). The relative abundance values of differential bacteria were extracted to correlate with ghrelin and glucose metabolic indices. Mediation analysis was conducted to evaluate ghrelin's effect on the relationship between bacterial abundance and glucose metabolic indices.

**Results:** Acylated ghrelin (AG) levels were significantly elevated in the T2DM group and showed positive correlations with glycated hemoglobin (HbA1c), fasting blood glucose (FBG), C-peptide (CP), and homeostasis model assessment of insulin resistance (HOMA-IR). Microbial analysis revealed decreased abundances of *Akkermansia*, *Lachnospira*, and *Phocaeicola*, but increased abundances of *Enterobacter*, *Escherichia*, *Megamonas*, *Segatella*, *Streptococcus*, and *Leyella* in T2DM. AG levels were negatively correlated with *Akkermansia* abundance. HbA1c and FBG showed positive correlations with *Enterobacter*, *Segatella*, and *Leyella* abundances, but negative correlations with *Akkermansia*, *Lachnospira*, and *Phocaeicola* abundances. HOMA-IR was positively correlated with *Segatella*, *Leyella*, and *Streptococcus* abundances, but negatively correlated with *Akkermansia* and *Lachnospira* abundances. Mediation analysis demonstrated that serum AG partially mediated the association between *Akkermansia* abundance and both HOMA-IR (26.15%) and HbA1c (27.91%).

**Conclusions:** AG mediated the relationship between *Akkermansia* abundance and HOMA-IR as well as HbA1c.

## 1. Introduction

Gut microbiota is a complex ecosystem containing a large amount of information. A growing number of evidences suggest that the gut microbiota has played a vital role in the development of Type 2 diabetes mellitus (T2DM) and has become a new target for alleviating hyperglycemia. The gut microbiota produces various metabolites including secondary bile acids, indole, water-soluble vitamins, short-chain fatty acids (SCFAs), and lipopolysaccharide (LPS), which influence sugar metabolism by affecting inflammation, immune response, and intestinal barrier integrity [1]. LPS derived from opportunistic gram-negative species can cause chronic low-grade inflammation, which is a key contributor involved in the onset of T2DM [2]. Instead, the production of SCFAs, including butyrate, propionate, and acetate, by commensal bacteria during dietary fiber fermentation has been shown to increase insulin sensitivity and decrease blood glucose concentrations [3]. The gut microbiota of diabetic patients shows a prevalence of opportunistic and pathogenic gram-negative species with a corresponding decrease in commensal ones.

In addition to the gut microbiota, gut hormones produced by enteroendocrine cells (EECs) also participate in the pathophysiology of T2DM. The gut microbiota can modulate the number of EECs, gut hormone biosynthesis, and secretion through its metabolites. Animal experiments observed that the gut microbiota regulated glucose homeostasis via glucagon-like peptide-1 (GLP-1) [4]. Besides GLP-1, as a gut hormone, ghrelin also has various effects on glucose metabolism, and its abnormal level is associated with T2DM. Ghrelin is primarily synthesized in the gastric mucosa by endocrine X/A cells (known as P/D1 cells in humans) and is acylated by ghrelin O-acyltransferase to produce acylated ghrelin (AG), which activates the ghrelin receptor [5]. AG injection in humans induces hyperglycemia and insulin resistance [6]. Moreover, AG exerts an inhibitory regulation on insulin secretion [7]. In ghrelin cells, a large number of receptors for metabolites of gut microbiota are expressed, especially G protein-coupled receptors for SCFAs, which are related to the suppression of ghrelin secretion [8]. Therefore, it can be inferred that gut microbiota may regulate blood glucose by modulating ghrelin secretion. However, studies on the relationship between gut microbiota and ghrelin remain limited.

This cross-sectional study aimed to identify gut microbiota taxa associated with ghrelin levels and clinical characteristics of T2DM. Mediation analyses were further performed to examine whether ghrelin levels mediated the associations between specific bacterial abundance and glucose metabolic indices, thereby exploring potential mechanisms of gut microbiota in blood glucose regulation.

## 2. Materials and Methods

### 2.1. Participants

Patients with newly diagnosed T2DM without using any hypoglycemic agents were recruited from the endocrinology department between November 2023 and April 2024. Subjects meeting one of the following criteria were excluded: (1) less than 18 years old or more than 60 years old; (2) history of chronic digestive system disorder; (3) history of diabetes chronic complications, including diabetic peripheral neuropathy, diabetic retinopathy, or diabetic nephropathy; (4) diabetic ketosis, hyperglycemic hyperosmolar status, severe hypoglycemia, and other acute complications; (5) history of psychiatric disorders; (6) history of cardiac, liver, or renal dysfunction; (7) history of anemia and other diseases that affect glycated hemoglobin (HbA1c); and (8) consumption of probiotics or antibiotics within 1 month prior to admission. Meanwhile, healthy controls (HCs) were selected from the physical examination center, whose age, gender, and dietary habits matched those of patients with T2DM. Each subject signed informed consent before inclusion. This study was censored and approved by the Medical Research Ethics Committee of The Seventh Affiliated Hospital, Sun Yat-sen University (KY-2023-105-01).

### 2.2. Information and Sample Collection

Detailed information on sociodemographic characteristics, dietary habits, lifestyles, disease and health conditions, medication usage, and dietary supplement intake were gathered through a comprehensive questionnaire. After fasting for 8 h, peripheral venous blood was taken in the morning. Fresh, middle, and inner fecal samples were collected from the participants using a special fecal sampler. All samples were promptly frozen in a refrigerator at −80°C until sequencing.

### 2.3. Biochemical Test

HbA1c was measured by high-performance liquid chromatography (VARIANT II TURBO 2.0 Glycosylated Hemoglobin Analyzer, Bio-Rad, United States). Fasting blood glucose (FBG) was determined by an automatic biochemical analyzer (Model C16000 Series Automatic Analyzer, Abbott, United States). C-peptide (CP) concentrations were tested by an automatic immunoassay analyzer (Model I2000 Series Automatic Analyzer, Abbott, United States). Insulin resistance was evaluated by improving the HOMA formula with fasting CP instead of insulin [9]. AG and unacylated ghrelin (UAG) were measured using the AG ELISA kit (ELabscience, Wuhan, Hubei Province, China) and the UAG ELISA kit (Yuanjie, Shanghai, China), respectively.

### 2.4. 16S rRNA Gene Amplification and Sequencing

Genomic DNA was extracted from each fecal sample using the MagPure Stool DNA KF Kit B (MAGEN, MD5115-02B) according to the manufacturer's instructions. For 16S amplicon sequencing, the hypervariable V3–V4 region of 16S rRNA was amplified with barcoded primers 338F (5⁣′-ACTCCTACGGGAGGCAGCAG-3⁣′) and 806R (5⁣′-GGACTACHVGGGTWTCTAAT-3⁣′), and the PCR products were then purified through agarose gel electrophoresis. Subsequently, the PCR products without contaminant bands and primer dimers were pooled in equimolar ratios and sequenced using the MGISEQ-2000RS (BGI, Shenzhen, China).

### 2.5. Meta-Amplicon Analysis

After sequencing, the data were merged, trimmed, and quality-filtered to generate high-quality clean data. The final clean data were clustered into operational taxonomic units (OTUs) at 97% similarity using USEARCH (Version 7.0.1090). Alpha diversity metrics (Ace, Shannon, Simpson, and Chao1 indices) were calculated with Mothur (Version 1.31.2). Beta diversity analysis was conducted using partial least squares discriminant analysis (PLS-DA) by QIIME (Version 1.8.0) to compare the microbial composition between samples. Prior to PLS-DA, microbial abundance data were normalized using cumulative sum scaling (CSS), with a quantile threshold of *q* = 0.5. Linear discriminant analysis effect size (LEfSe) was used to identify differentially abundant taxa across groups, with a significance threshold of LDA score > 3.0 (Kruskal–Wallis test, *p* < 0.05; Benjamini–Hochberg corrected *q* < 0.1). Finally, the relative abundance values of the Top 10 differential species were extracted for further analysis.

### 2.6. Statistical Analysis

Continuous data were presented as mean ± standard deviation (normally distributed) or median with interquartile range (25th–75th percentiles; nonnormally distributed). Normality was assessed using the Shapiro–Wilk test, and homogeneity of variance was verified via Levene's test. Comparisons between groups were performed using Student's *t*-test for normally distributed variables, the Mann–Whitney *U* test for nonnormally distributed variables, and the chi-squared test for categorical variables. Spearman's rank correlation analysis was used to evaluate correlations between differential bacterial abundance, AG levels, and biochemical indices.

Mediation analysis was performed to examine the mediating effect of AG levels between differential bacterial abundance and biochemical indices, with age, sex, and body mass index (BMI) as covariates. The mediation effect (ME) was quantified as the indirect effect of the independent variable (*X*; bacterial abundance) on the dependent variable (*Y*; glucose metabolic indices) through the mediator (*M*; AG). This effect was calculated as the product of two regression coefficients: *a* = *e*ffect of *X* on *M* (regression coefficient: *X*⟶*M*), *b* = Effect of *M* on *Y* while controlling for *X* (regression coefficient: *M*⟶*Y*/*X*), ME = *a* × *b*. Statistical significance of the indirect effect was determined using bootstrap resampling (5000 iterations) with 95% confidence intervals (CI). A ME was considered significant if the CI excluded zero.

Statistical analysis was executed by SPSS software (Version 22) and R software (Version 4.1.2). A two-sided *p* value of 0.05 was considered statistically significant. Multiple comparisons were adjusted using the Benjamini–Hochberg method, and a threshold of false discovery rate (FDR) adjusted *p* value of 0.05 was used.

## 3. Results

### 3.1. Demographic and Clinical Characteristics

A total of 55 T2DM and 40 HC participants were enrolled in this study. The demographic and clinical characteristics are presented in Table 1. No significant differences were observed in age, sex, and BMI between the two groups. However, the T2DM group exhibited significantly higher levels of HbA1c, FBG, CP, and HOMA-IR compared to the HC group. Furthermore, AG levels were significantly elevated in the T2DM group relative to the HC group, whereas UAG levels showed no significant intergroup difference.

### 3.2. Correlations Between AG and Glucose Metabolic Indices

**Figure 1:**
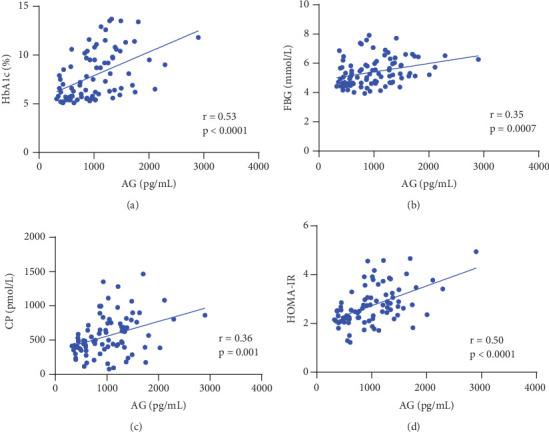
Correlation between AG and glucose metabolic indices. (a) Correlation between AG and HbA1c. (b) Correlation between AG and FBG. (c) Correlation between AG and CP. (d) Correlation between AG and HOMA-IR. Abbreviations: AG, acylated ghrelin; HbA1c, hemoglobin A1c; FBG, fasting blood glucose; CP, C-peptide; HOMA-IR, homeostasis model assessment of insulin resistance.

As shown in Figure 1, AG was positively correlated with HbA1c, FBG, CP, and HOMA-IR, with the correlation coefficient being 0.49, 0.46, 0.36, and 0.50, respectively (all *p* values ≤ 0.001).

### 3.3. Gut Microbiota Distribution and Diversity

**Figure 2:**
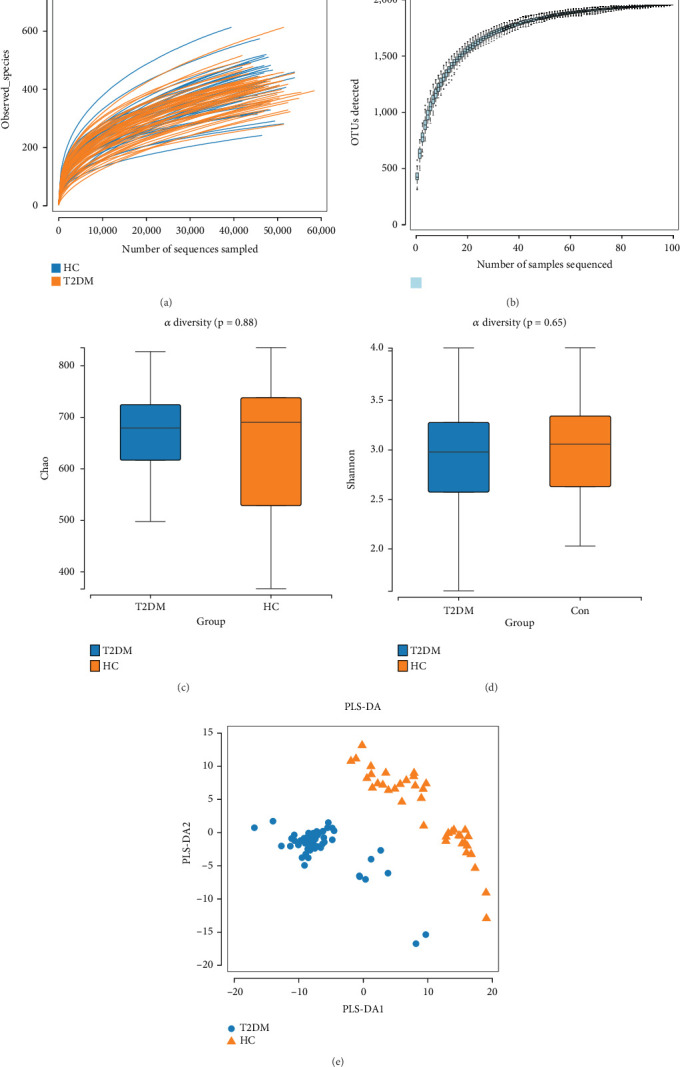
Gut microbiota alpha and beta diversity indices in T2DM and HC. (a) The rarefaction curve. (b) The species accumulation curve. (c) The Chao1 index between groups. (d) The Shannon index between groups. (e) The partial least squares discriminant analysis (PLS-DA) between groups.

The rarefaction curve exhibited progressive flattening, confirming that our sequencing depth adequately captured the microbial community diversity (Figure 2a). Similarly, the species accumulation curve showed a flattening trend with increasing sample size, indicating sufficient sampling coverage (Figure 2b). Alpha diversity was assessed using both Chao1 (community richness) and Shannon (community diversity) indices. As shown in Figure 2c,d, no significant differences in alpha diversity were observed between groups. For beta diversity analysis, PLS-DA revealed distinct clustering patterns, demonstrating significant differences in gut microbiota composition between the T2DM and HC groups (Figure 2e).

### 3.4. Bacteria With Differential Abundance Between T2DM and HC

**Figure 3:**
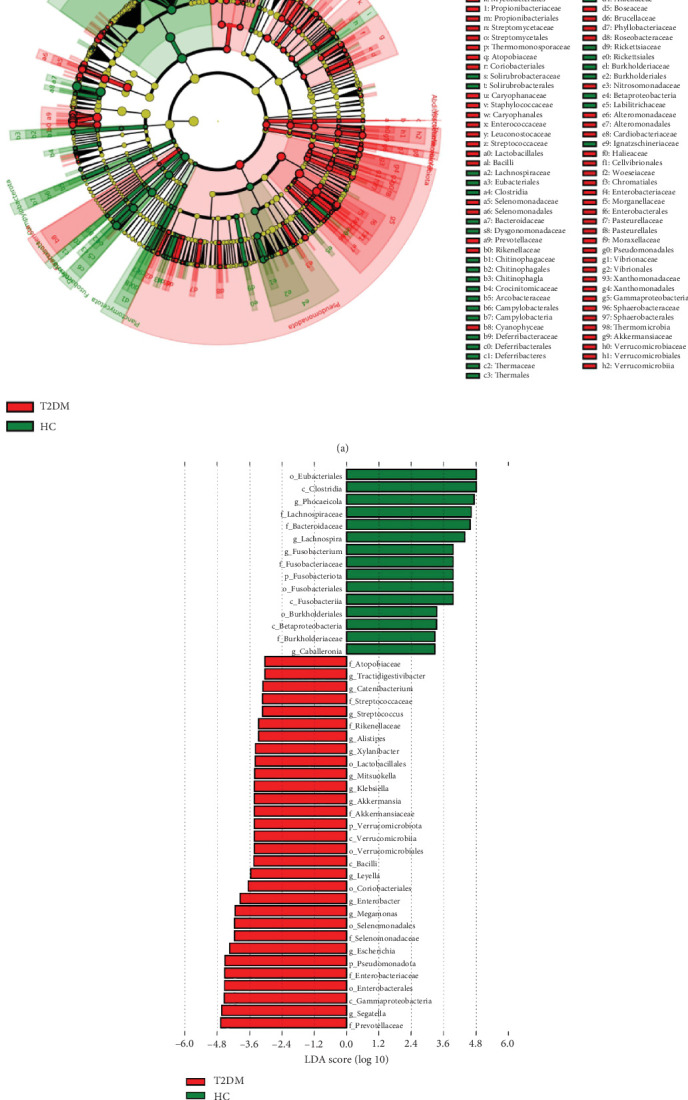
LEfSe analysis to determine bacteria with differential abundance between groups. (a) LEfSe clustering plot. (b) LEfSe LDA plot. The absolute value of LDA score greater than 3 is shown in the figure. Abbreviation: LEfSe, linear discriminant analysis effect size.

**Figure 4:**
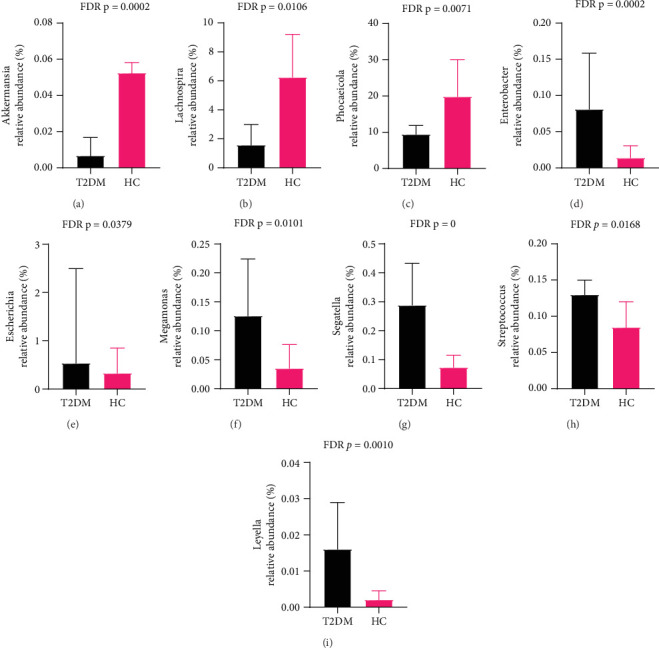
(a–i) Bacteria with differential abundance at the genus level. Abbreviation: FDR *p*, false discovery rate adjusted *p* value.

To identify the microbe difference between the T2DM and HC groups, LEfSe analysis was performed (Figure 3). We showed the Top 10 differential species with different abundances. At the genus level, the abundance of *Akkermansia*, *Lachnospira*, and *Phocaeicola* decreased, while the abundance of *Enterobacter*, *Escherichia*, *Megamonas*, *Segatella*, *Streptococcus*, and *Leyella* increased in the T2DM group compared with the HC group (FDR *p* < 0.05, Figures 4a, 4b, 4c, 4d, 4e, 4f, 4g, 4h, and 4i).

### 3.5. Correlations Between Biochemical Indices and Gut Microbiota

**Figure 5:**
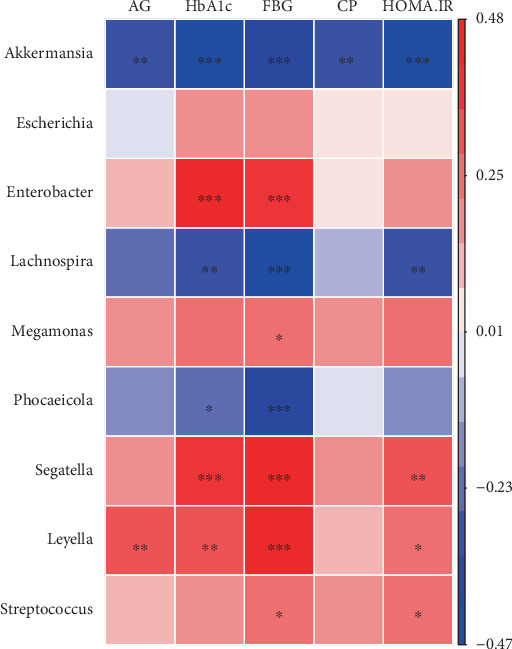
Heatmaps of Spearman's correlations between differential bacteria abundance, AG concentration, and glucose metabolic indices. ⁣^∗^*p* < 0.05, ⁣^∗∗^*p* < 0.01, and ⁣^∗∗∗^*p* < 0.001. Abbreviations: AG, acylated ghrelin; HbA1c, hemoglobin A1c; FBG, fasting blood glucose; CP, C-peptide; HOMA-IR, homeostasis model assessment of insulin resistance.

Correlation analyses were performed between clinical parameters and differentially abundant bacterial taxa. AG showed a negative correlation with *Akkermansia* abundance and a positive correlation with *Leyella* abundance. HbA1c was positively correlated with *Enterobacter*, *Segatella*, and *Leyella* abundances, but negatively correlated with *Akkermansia*, *Lachnospira*, and *Phocaeicola* abundances. Similarly, FBG exhibited positive correlations with *Enterobacter*, *Megamonas*, *Segatella*, *Leyella*, and *Streptococcus* abundances while showing negative correlations with *Akkermansia*, *Lachnospira*, and *Phocaeicola* abundances. HOMA-IR demonstrated positive associations with *Segatella*, *Leyella*, and *Streptococcus* abundances and negative associations with *Akkermansia* and *Lachnospira* abundances. These correlation patterns are visualized in the heatmap presented in Figure 5.

### 3.6. MEs Between *Akkermansia* Abundance and Metabolic Indices via AG

**Figure 6:**
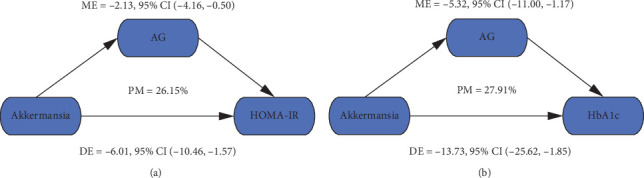
AG mediated the association between *Akkermansia* abundance and glucose metabolic indices. (a) ME between *Akkermansia* abundance and HOMA-IR. (b) ME between *Akkermansia* abundance and HbA1c. Abbreviations: AG, acylated ghrelin; HbA1c, hemoglobin A1c; HOMA-IR, homeostasis model assessment of insulin resistance; ME, mediation effect; DE, direct effect; PM, proportion mediated.

The abundance of *Akkermansia* showed significant correlations with both AG levels and glucose metabolic indices. We conducted mediation analyses treating *Akkermansia* abundance as the independent variable, glucose metabolic indices (HOMA-IR, FBG, and HbA1c) as dependent variables, and AG as the mediator while adjusting for age, sex, and BMI.  Figure 6a,b shows the MEs between *Akkermansia* abundance and HOMA-IR and HbA1c via AG. Serum AG level significantly and partially mediated the association between *Akkermansia* abundance and HOMA-IR and HbA1c, with mediating effects of 26.15% and 27.91%, respectively (all *p* values < 0.001). However, serum AG level did not mediate the association between *Akkermansia* abundance and FBG.

## 4. Discussion

This study not only demonstrated significant negative correlations between *Akkermansia* abundance and key metabolic parameters (AG, HbA1c, FBG, CP, and HOMA-IR) but also revealed that AG partially mediated the *Akkermansia*-glucose homeostasis relationship, accounting for 26.15% and 27.91% of the effects on HOMA-IR and HbA1c, respectively. These findings align with the growing application of mediation analysis in biomedical research, which provides valuable insights into potential causal pathways beyond simple associations. Particularly in microbiome studies, this approach helps bridge the gap between microbial compositional changes and their functional metabolic consequences. Our results suggest that *Akkermansia* may influence glucose homeostasis through both AG-dependent and independent mechanisms, offering new perspectives for developing microbiota-targeted interventions in T2DM management.

Since *Akkermansia muciniphila* was discovered and identified 20 years ago, many studies have shown that the decreased abundance of this symbiotic bacterium is closely related to many metabolic diseases, especially diabetes and obesity. Belonging to the Verrucomicrobiae phylum and *Akkermansiaceae* family, *Akkermansia* is a gram-negative, strictly anaerobic, mucin-degrading bacterium [10]. As a mucin-degrading specialist, *Akkermansia* would interact preferentially with intestinal cells and affect health, because supplementing *Akkermansia* can increase the number of mucus-producing cells [11]. Increasing studies have found that the abundance of *Akkermansia* decreased in both T2DM animals and patients [12]. Several animal experiments and clinical trials have reported that *Akkermansia* administration could improve insulin sensitivity and reduce insulinemia and glucose [13, 14]. Although increasing data have proved the benefits of *Akkermansia* on T2DM, the underlying mechanisms remain obscure.

By reducing LPS and gut permeability, *Akkermansia* alleviates metabolic endotoxemia, which is the initial factor of diabetes [15]. In addition, *Akkermansia* degrades mucin and produces some bioactive metabolites such as SCFAs, indole derivatives, and polysaccharides [16]. However, how these metabolites play a role in improving metabolism is still unclear. It was demonstrated that *Akkermansia* treatment exhibited an increase in circulating GLP-1 levels during an oral glucose challenge in diet-induced obesity mice [17]. After bariatric surgery, it is not only the increase of GLP-1 but the decrease of ghrelin that is responsible for the beneficial effects in diabetes and obesity [18]. As an indispensable intestinal hormone, ghrelin is very vital for the development of T2DM. However, how *Akkermansia* affects the level of ghrelin is still unknown.

Our mediation analysis reveals that AG may serve as a target for *Akkermansia* to ameliorate insulin resistance. Given the presence of SCFA receptors on ghrelin-producing cells, we hypothesize that *Akkermansia*-derived SCFAs could directly modulate ghrelin secretion. These findings suggest potential therapeutic applications. Pharmacological modulation of ghrelin signaling (e.g., AG antagonists or ghrelin receptor blockers) could help mitigate insulin resistance and hyperglycemia, particularly in individuals with low *Akkermansia* levels.

A diverse array of microbial taxa colonizes the human gastrointestinal tract, forming a complex ecosystem through intricate ecological interactions. These microbial communities engage in dynamic competition and cooperation, significantly influencing host metabolic health. In addition to *Akkermansia*, our study identified several differentially abundant bacterial taxa whose abundances correlated with glycemic parameters (HbA1c, FBG, and HOMA-IR). Previous studies have demonstrated that the abundance of *Enterobacteriaceae*, *Clostridiales*, *Lactobacilli*, *Prevotella copri*, and other conditional pathogens increased, while the abundance of SCFA-producing bacteria such as *Bifidobacterium*, *Bacteroides*, *Akkermansia muciniphila*, and *Roseburia* decreased in T2DM patients, which is highly consistent with our research [19].

At the genus level, we observed significantly higher abundances of *Enterobacter*, *Escherichia*, *Megamonas*, *Segatella*, *Streptococcus*, and *Leyella* in the T2DM group compared to HC. Further correlation analysis revealed that the abundances of *Enterobacter*, *Segatella*, and *Leyella* were positively correlated with HbA1c levels; the abundances of *Enterobacter*, *Megamonas*, *Segatella*, *Leyella*, and Streptococcus were positively correlated with FBG; and the abundances of *Segatella*, *Leyella*, and *Streptococcus* were positively correlated with HOMA-IR. Both *Enterobacter* and *Escherichia* belong to the *Enterobacteriaceae* family, which causes intestinal endothelial cell damage, leading to infectious diarrhea and other opportunistic infections. It has been shown that severe infection is related to the increase of new-onset diabetes, and the nosogenesis is related to *β*-cell death caused by persistent inflammation and fibrosis [20]. Belonging to the phylum Firmicutes, *Megamonas* can use a variety of carbohydrates to produce acidic compounds, which are substrates for fat formation, cholesterol formation, and gluconeogenesis [21]. A recent study also showed that *Megamonas* can enhance lipid absorption and obesity by degrading inositol [22]. Therefore, *Megamonas* has been consistently overrepresented in individuals with obesity, diabetes, and nonalcoholic fatty liver disease across multiple studies [23]. *Segatella* and *Leyella*, which belong to the family *Prevotellaceae*, have also been associated with inflammatory conditions—a key pathological feature of obesity and metabolic diseases.

In addition to *Akkermansia*, the abundances of *Lachnospira* and *Phocaeicola* were also decreased in the T2DM group at the genus level. *Lachnospira*, a butyrate-producing genus within the Firmicutes phylum, contributes to intestinal barrier integrity through multiple mechanisms by maintaining tight junctions and epithelial homeostasis [24]. *Phocaeicola* belongs to *Bacteroidota*; it has been shown to decrease in the gut microbiome of patients with coronary artery disease. Oral administration of *Phocaeicola* can decrease the fecal and plasma LPS concentrations and protect mice against atherosclerosis [25]. Notably, high-fat-diet-fed mice treated with *Phocaeicola* showed improved metabolic parameters (reduced body weight, insulin resistance, and intestinal permeability) alongside restored *Akkermansia* abundance [26]. These findings suggest that cooperative interactions among these bacterial taxa may enhance insulin sensitivity and mitigate metabolic disease progression.

There are several limitations in this study. First, as a cross-sectional study, our findings are inherently limited in establishing causal relationships. Although mediation analysis can help explore potential effect pathways, its results in cross-sectional designs should be interpreted with caution due to the lack of temporal sequence evidence. Further longitudinal or interventional studies are required to validate causality. Additionally, animal experiments need to be conducted to observe the effects of *Akkermansia* gavage on ghrelin levels and glucose metabolism. Secondly, compared with metagenome sequencing, 16S rRNA sequencing cannot provide detailed information at the strain level and cannot conduct in-depth research on genes and functions.

## 5. Conclusion

In conclusion, this study explored the potential relationships between gut microbiota (particularly *Akkermansia*), ghrelin, and insulin resistance. Our mediation analysis suggested that serum AG might play a mediating role in the observed associations between *Akkermansia* abundance and both HOMA-IR and HbA1c. These preliminary findings raise the possibility that modulation of AG secretion could represent one potential mechanism through which *Akkermansia* might influence insulin sensitivity, though further mechanistic studies are needed to confirm this hypothesis.

## Figures and Tables

**Table 1:** Demographic and clinical characteristics of T2DM and HC.

**Characteristics**	**T2DM (** **n** = 55**)**	**HC (** **n** = 40**)**	**p** ** value**
Age (years)	50.61 ± 12.28	47.29 ± 9.11	0.20
Sex (F/M)	19/36	18/22	0.30
BMI (kg/m^2^)	24.74 ± 3.73	24.30 ± 2.65	0.57
HbA1c (%)	9.20 (7.50, 10.80)	5.5 (5.30, 5.60)	< 0.001
FBG (mmol/L)	7.01 (6.37, 9.53)	4.94 (4.62, 5.31)	< 0.001
CP (pmol/L)	619.26 (393.02, 831.50)	475.95 (368.70, 588.70)	0.045
HOMA-IR	3.06 (2.54, 3.84)	2.28 (2.10, 2.55)	< 0.001
AG (pg/mL)	1193.35 (872.41, 1440.02)	743.49 (544.10, 998.08)	0.001
UAG (pg/mL)	973.78 (856.44, 1213.98)	922.11 (858.89, 1048.70)	0.23

Abbreviations: AG, acylated ghrelin; BMI, body mass index; CP, C-peptide; F, female; FBG, fasting blood glucose; HbA1c, hemoglobin A1c; HCs, healthy controls; HOMA-IR, homeostasis model assessment of insulin resistance; M, male; T2DM, Type 2 diabetes mellitus; UAG, unacylated ghrelin.

## Data Availability

The data that support the findings of this study are available from the corresponding authors upon reasonable request.
